# Nebulized glycosylated caffeic acid phenylether ester attenuation of environmental particulate-induced airway inflammation in horses

**DOI:** 10.3389/fvets.2022.958567

**Published:** 2022-11-03

**Authors:** Jessica J. Rutledge, Jillian Paegelow, Jerry Ritchey, Anuradha Singh, Theresa Rizzi, Cynthia Murray, Lyndi Gilliam, Evan Crisman, Natasha J. Williams, Todd C. Holbrook

**Affiliations:** ^1^Department of Veterinary Clinical Sciences, Oklahoma State University, Stillwater, OK, United States; ^2^Department of Veterinary Pathobiology, Oklahoma State University, Stillwater, OK, United States; ^3^Department of Chemistry, Oklahoma State University, Stillwater, OK, United States; ^4^Department of Mathematics and Statistics, University of Central Oklahoma, Edmond, OK, United States

**Keywords:** equine asthma, neutrophilia, airway inflammation, nebulization, particulates, hay, horses

## Abstract

The objective of this study was to determine the extent that nebulized glycosylated caffeic acid phenylether ester-4-O-alpha-D-glucopyranoside (G-CAPE) attenuates particulate-induced airway inflammation in healthy horses. Our hypothesis was that nebulization with G-CAPE would result in improved respiratory scores, higher arterial oxygen partial pressure, and less inflammatory airway infiltrates in horses with induced airway inflammation, compared with untreated controls. Five healthy adult horses were housed inside a climate controlled, closed barn on straw bedding and fed *ad lib* moldy grass hay for 16 days to induce airway inflammation. An experimental crossover study was performed in which animals were treated with 200 mg G-CAPE dissolved in 45 mL of 10% triethanolamine (G-CAPE group) or 45 mL of 10% triethanolamine (CONTROL group), and clinical respiratory scoring, arterial blood gases, and bronchoalveolar lavages (BALs) were collected at predetermined time points up to 24 h post nebulization. While the mean neutrophil percentage decreased in treated horses compared to controls (9.3 ± 2.0 and 16.9 ± 2.4, respectively) at 6 hours post treatment (*t* = 6 h), the difference did not achieve statistical significance (*p* = 0.1154). Blood gas analysis did not differ significantly between groups. There was a significant difference in the mean respiratory scores of G-CAPE-treated horses between baseline and at 1-h post treatment (from 3.2 ± 0.7 to 1.6 ± 0.7, *p* = 0.0013). This study demonstrates that a single nebulized dose of G-CAPE decreased clinical respiratory scores 1 h post administration and decreased BAL percentage of neutrophils 6 h post administration in horses with particulate induced airway inflammation. This compound shows promise as an anti-inflammatory and warrants further investigation.

## Introduction

Equine asthma is a group of chronic, non-septic, inflammatory airway diseases previously known as either inflammatory airway disease (IAD), recurrent airway obstruction (RAO), or summer pasture-associated recurrent airway obstruction (1, 2). These conditions are now classified as equine asthma, and further subdivided into mild-moderate and severe equine asthma. Lower airway inflammation consistent with mild equine asthma has been detected in 66–80% of horses and both mild and moderate equine asthma are common causes of poor performance (3, 4). Severe equine asthma affects a smaller percentage of horses (10–20%) but may interfere with resting respiratory function in addition to affecting their athletic activity (5, 6).

Whilst the mainstay of current equine asthma treatments consists of environmental management, including limiting dust and mold exposure, the addition of bronchodilators and systemic or inhaled corticosteroids are often required as adjunctive therapy. Long term corticosteroid use is known to have significant side effects in horses, including hepatopathy, susceptibility to infections, viral recrudescence, hyperglycemia, insulin resistance, and hyperinsulinemia (7–11). Hyperinsulinemia is problematic, as it can lead to the development of laminitis, especially in susceptible populations of horses and ponies affected with pituitary pars intermedia dysfunction or equine metabolic syndrome (12). Inhaled corticosteroid therapy carries a reduced risk of side effects, but may incur significant financial cost (13, 14). There is a critical need for an alternative anti-inflammatory treatment other than corticosteroids that are safe and cost-effective for the long- term treatment of asthmatic horses.

Nuclear factor kappa B (NF-κB) is a transcription factor vital to the activation of multiple proinflammatory genes (15) and amplification of the inflammatory response through production of cytokines, enzymes, adhesion molecules, and acute phase proteins (16). NF-κB is a key factor in the pathophysiology of asthma and an ideal target for therapy (17, 18). Caffeic acid phenylethyl ester (CAPE) is an antioxidant compound found in propolis of honeybee hives and has been demonstrated to be a specific inhibitor of NF-κB activation (16, 19). Mice with experimentally-induced asthma treated with CAPE demonstrated less inflammatory cell infiltration, cytokine production, mucus secretion, collagen deposition, and fibrosis than did non-treated controls (20). Cytokines noted to be increased in the bronchoalveolar lavage (BAL) fluid of asthmatic horses include TNF-α and IFN-γ (21); these provide appealing therapeutic targets to disrupt pulmonary inflammation and neutrophil recruitment in equine asthma.

CAPE's promise as a useful therapeutic has been limited by its poor solubility in aqueous solution. However, several studies have shown that glycosylation of the molecule can overcome these limitations. The glycosylated compound, caffeic acid phenylether ester-4-O-alpha-D-glucopyranoside (G-CAPE), is a pro-drug metabolized into CAPE within cells (22). G-CAPE has been found to be more stable, 770 times more soluble than CAPE (23), and a more potent anti-inflammatory (24, 25). Improved solubility and enhanced anti-inflammatory properties support G-CAPE's promise as a viable therapeutic for equine asthma.

The objective of this study was to determine the extent that nebulized glycosylated CAPE (G-CAPE) attenuates particulate-induced airway inflammation in healthy horses. In previous studies using mice models, the route of administration utilized was intraperitoneal injection (16, 26). The nebulized route was chosen to target pulmonary inflammation, minimize the dose required, and limit any potential systemic side effects of G-CAPE absorption. We hypothesized that horses treated with G-CAPE would have a significant decrease in BAL neutrophils, improvement in clinical respiratory scores, and increased arterial oxygenation compared to controls.

## Materials and methods

### Animals

**Figure 1:**
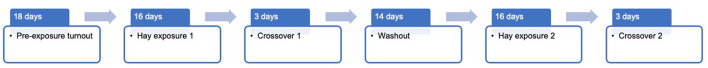
Timeline of experimental periods. Horses were housed together on the same pasture for 18 days, before being moved to a climate-controlled closed barn for the duration of the experimental procedures. Horses were returned to the same pasture for the 14-day washout period.

This study was approved by the Oklahoma State University Institutional Animal Care and Use Committee (Animal Care and Use Protocol IACUC-20-08) and carried out with strict adherence to all guidelines. Five clinically healthy adult horses (5–25 years) from a research herd were used in these studies. Horses were of different breeds (2 thoroughbreds, 2 Quarter Horses, 1 Oldenburg), sexes (1 mare, 4 geldings), and weights (range 477–634 kg). The study animals had no evidence of infectious disease observed on physical exam, rebreathing examination, complete blood count, or serum biochemistry (including fever (>38.3C) or significant bloodwork abnormalities). Prior to the experiment, all horses were housed together on the same pasture for 18 days. During the experimental periods (16-day exposure and 3-day treatment crossover periods), horses were housed inside a climate controlled closed barn on straw bedding (see Figure 1). The extractor/air exchange fan was run for 4 h per day, and the stalls were cleaned once daily. Whilst in the barn, the horses were fed ad lib moldy grass hay, sectioned from a round bale, in a plastic 55-gallon drum on the ground with the top removed to encourage them to keep their noses in the drum for prolonged periods of time in order to increase particulate exposure.

### G-CAPE synthesis

Caffeic acid phenylether ester-4-O-alpha-D-glucopyranoside was synthesized in three steps as previously described in the literature (27). Briefly, alkylation of caffeic acid with 2-(bromoethyl) benzene in the presence of sodium carbonate and catalytic potassium iodide in dimethylsulfoxide was performed to procure caffeic acid phenylether ester (CAPE). The resultant compound was purified by recrystallization in hexane and ethyl acetate, and the structure of the CAPE was confirmed by matched proton nuclear magnetic resonance (NMR) spectrum (27). Glycosylation of CAPE was accomplished using 2,3,4,6-tetra-O-acetyl-alpha-D-glucopyranosyl bromide in dichloromethane (28). Ethyl 4-(2′,3′,4′,6′-tetra-O-acetyl-alpha-D-glucopyranosyl) caffeate was isolated by silica gel column chromatography using eluents hexane and ethyl acetate. Finally, deacetylation was executed using catalytic sodium methoxide in methanol followed by silica gel column chromatography purification using ethyl acetate and methanol to acquire G-CAPE. The structure of G-CAPE was confirmed by matched literature's proton and carbon NMR spectrum (22, 23).

### Clinical respiratory scoring

Clinical respiratory scores were performed by one of two trained DVMs using the subjective clinical scoring system “Improved clinically Detectable Equine Asthma Scoring System” (IDEASS) described by Calzetta et al., adapted from Rush et al. (2, 29). Briefly, each horse was given a score of 0–4 corresponding to the abdominal component (0 = normal, 1 = slight abdominal effort, 2 = moderate abdominal effort, 3 = severe abdominal effort, 4 = very severe abdominal effort) and nostril flare (0 = normal, 1 = slight occasional flare, 2 = moderate and occasional flare, 3 = severe and persistent flare, 4 = very severe and persistent flare); the two scores were totaled to quantify induced asthma severity on a scale of 0–8 (0 = normal, 1–2 = mild, 3–4 = moderate, 5–6 = severe, 7–8 = very severe).

### Treatment crossover

A randomizer was used to determine which horses would receive the G-CAPE in crossover one. Prior to nebulization, horses underwent physical examinations, clinical respiratory scoring, blood gas analysis, and BAL. Blood gas analysis (CG8+ cartridge, iStat1, Abbot) was performed stall-side using blood collected from the transverse facial artery with a heparinized 1 mL syringe and a 22G ¾” needle (non-temperature corrected). BAL was performed under standing sedation with 10 μg /kg detomidine hydrochloride (Zoetis), i.v., After a period of at least 1-h post-sedation, horses received a single treatment utilizing an equine nebulizer (Flexineb, Nortev, Galway, Ireland) to administer a solution of either 200 mg G-CAPE dissolved in 45 mL of 10% triethanolamine (G-CAPE Group) or 45 mL of 10% triethanolamine (CONTROL group) (both solutions manufactured by Weaver labs, Stillwater, OK). The nebulizer was used to deliver the 45 mL of solution and was removed when all the volume was distributed without a standard exposure. It was cleaned between each horse according to the manufacturer's instruction.

**Figure 2:**
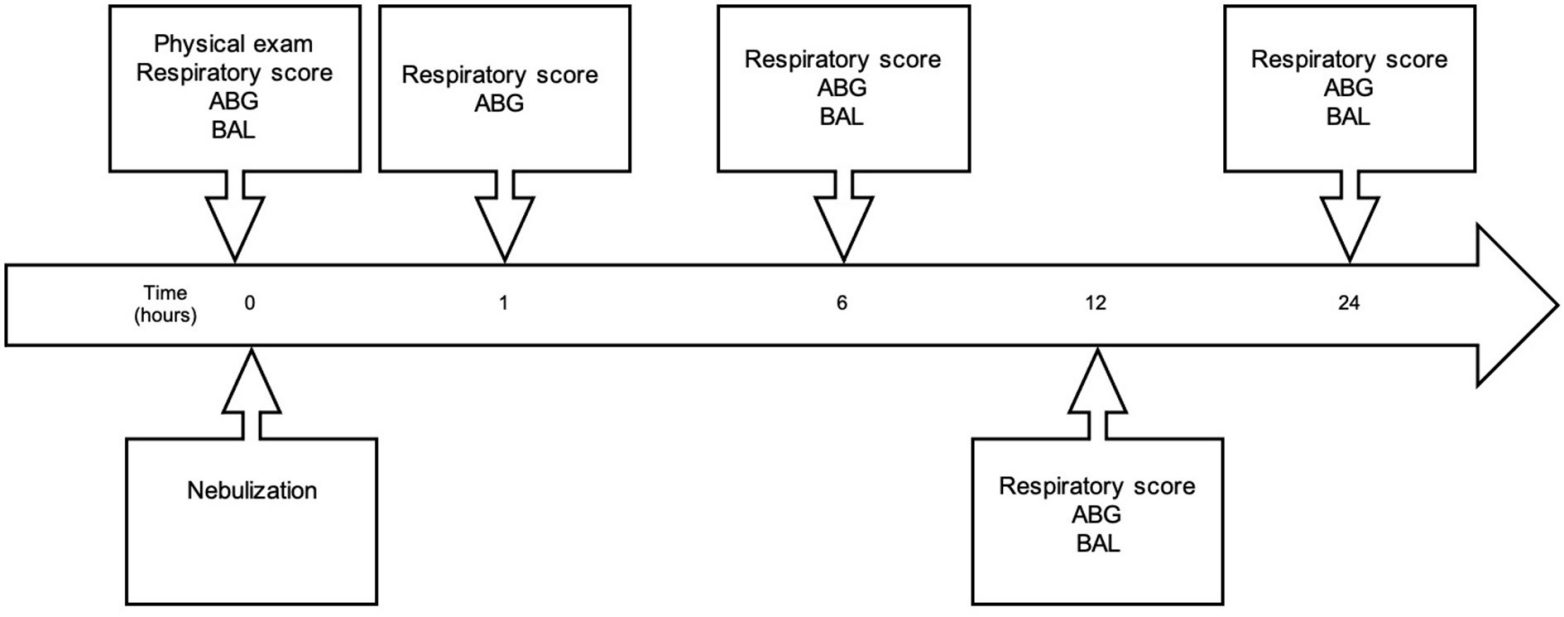
Timeline of experimental sampling. Immediately prior to nebulization, horses underwent physical examinations and arterial blood gas (ABG) sampling, and received baseline respiratory scores and bronchoalveolar lavage (BAL). Nebulization was then performed, and respiratory scores and ABG were repeated 1-h post completion of nebulization. Respiratory scores, ABG analysis, and BAL were repeated at 6-, 12- and 24-h post nebulization.

Respiratory scoring and arterial blood gas analyses were repeated at 1, 6, 12, 24 h post treatment (see Figure 2). BAL was repeated at 6 and 24 h post treatment. After a 14-day washout period at pasture, the horses were returned to the barn for the second 16-day hay exposure, before receiving the alternative treatment (G-CAPE or CONTROL) in crossover two.

### BAL sampling and analysis

Each procedure was performed using a 3-meter long, 8-mm diameter flexible video endoscope (Olympus) and alternating between right and left bronchi to minimize the confounding effect of BAL-induced inflammation. No attempt was made to return to the same bronchus when returning to the same side. The distal trachea was instilled with 20 mL of 2% lidocaine to minimize coughing, the scope advanced and wedged in a bronchus, and 240 mL of warm 0.9% saline (pre-incubated in a 37°C warm-water bath) was instilled through the biopsy channel. BAL fluid samples were recovered by manual aspiration, pooled in sterile glass bottles, immediately aliquoted into EDTA tubes, and refrigerated at 4°C until fluid analysis could be performed (within 3 h).

A blinded, board-certified clinical pathologist (TER) performed all BAL fluid analyses. Manual cell counts were performed using a Reichert Bright-Line^TM^ hemacytometer (Hausser Scientific, Horsham, PA, USA). Undiluted BAL fluid was loaded onto the hemacytometer where the total number of cells in all 9 squares of the counting chamber were counted. Both sides of the hemacytometer chamber were counted. The total cell count was obtained by dividing the average number of cells from the hemacytometer count by 0.9 (no diluent used). A cytocentrifuged preparation of the BAL fluid was stained with Wright-Giemsa and a 500-cell differential was performed. The nucleated cell count included macrophages, lymphocytes, eosinophils, neutrophils, mast cells, columnar epithelial cells, and goblet cells.

### Statistical analysis

The data was analyzed using ANOVAs, two-factor repeated measure design, for testing differences between the multiple time means and differences between the two group means (G-CAPE and CONTROL). Tukey's multiple comparison tests were used to determine which pairs of means, of all possible pairs, were significant. Measurements at times 0, 6, and 24 h, for each group of horses, included BAL total nucleated cell count, percent macrophages, macrophage count, percent lymphocytes, lymphocyte count, percent eosinophils, eosinophil count, percent neutrophils, neutrophil count, percent mast cells, mast cell count, percent goblet cells, goblet cell count, percent columnar cells, and columnar cell count. Measurements at times 0, 1, 6, 12, and 24 h, for each group of horses, included respiratory score, pO_2_, and pCO_2_. *P*-values < 0.05 were considered significant. All analyses were performed using SAS, MIXED procedure (version 9.4).

## Results

The insolubility of native CAPE severely impacts delivery and therapeutic potential. The glycosylation of the CAPE significantly improved solubility and permitted delivery by nebulization without drug precipitation. The treatment doses of 200 mg G-CAPE were dissolved in 45 mL of 10% triethanolamine and remained soluble in solution for up to 5 days when stored in airtight plastic containers at room temperature away from direct sunlight. The solution was nebulized with no difficulty, and all animals tolerated it well. Mean nebulization time for the treatment group was 56 min (range 42–74 min), whilst mean nebulization time for the control group was 51 min (range 31–79 min). Treatment and control animals were monitored throughout nebulization for negative effects (increased respiration rate, coughing). No animals displayed any of these signs, and body condition was maintained during the study.

**Figure 3:**
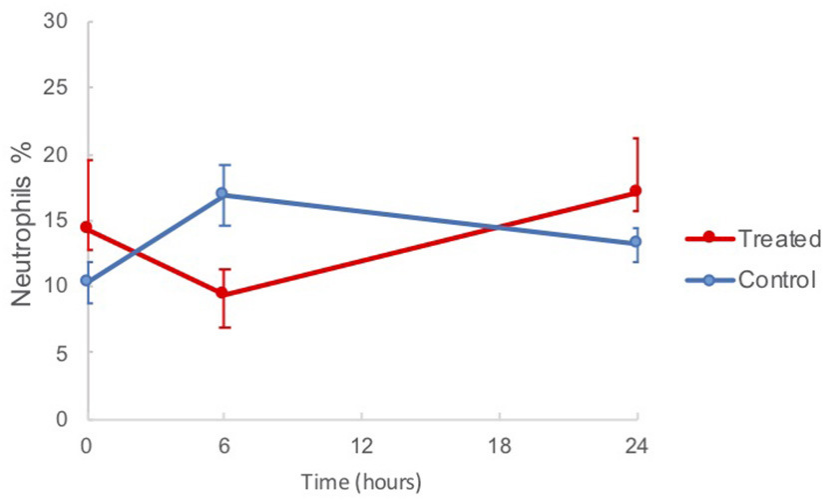
Mean BAL neutrophil percentages in horses from baseline (*t* = 0 h) to 24 h post nebulization with either control substance or G-CAPE (treated).

The results of the BAL fluid cell analyses are shown in Table 1. There was a reduction in the mean neutrophil percentage of treated horses (9.3 ± 2.0) compared to controls (16.9 ± 2.4) at 6 h post treatment, as shown in Figure 3, however the difference was not statistically significant (*p* = 0.1154). Compared to the baseline, at 6 h post nebulization, the total neutrophil count decreased in the treatment group (63.9 ± 24.0 to 46.4 ± 14.8), and was significantly increased in the control group (47.0 ± 7.0 to 84.8 ± 15.6, *p* = 0.0357). No signs of trauma were noted during repeat endoscopy.

**Table 1:** Grouped BAL results from baseline (*t* = 0 h) to 24 h post nebulization with either control substance (C) or G-CAPE (T), expressed as mean ± SE.

		**Time (hours)**					
		**0**		**6**		**24**	
		**C**	**T**	**C**	**T**	**C**	**T**
TNCC	/uL	484.3 ± 68.6	452.8 ± 31.2	535.6 ± 104.2	478.6 ± 61.5	573.8 ± 50.9	536.6 ± 47.1
Macrophages	%	54.9 ± 3.2	48.8^a^ ± 4.1	40 ± 2.8	44.8^a^ ± 2.6	53.1 ± 2.6	48.2^a^ ± 2.8
	/uL	261.2 ± 32.9	221.2 ± 25.7	220.8 ± 48.3	213.2 ± 26.3	304.8 ± 30.4	254.3 ± 30.7
Lymphocytes	%	30.6 ± 3	32.9 ± 3.5	37.1^c^ ± 2.7	40^b^ ± 1.4	29.4^c^ ± 2.3	30.7^b^ ± 3.5
	/uL	155.0 ± 34.5	150.6 ± 22.7	200.4 ±4 3.8	192.3 ± 28.6	171.2 ± 24.2	168 ± 75.1
Neutrophils	%	10.3^e^ ± 1.6	14.3 ± 5.3	16.9^d, e^ ± 2.4	9.3^d^ ± 2.0	13.2 ± 1.3	17.0 ± 4.2
	/uL	47.0 ± 7.0	63.9 ± 24.0	84.8 ± 15.6	46.4 ± 14.8	73.9 ± 6.9	92.4 ± 23.4
Eosinophils	%	0.4 ± 0.3	0.1 ± 0.1	0.4 ± 0.1	0.7 ± 0.4	0.2 ± 0.2	0.4 ± 0.3
	/uL	2.6 ± 1.9	0.6 ± 0.2	2.7 ± 1.1	3.9 ± 2.1	1.3 ± 0.8	2.6 ± 1.6
Mast cells	%	2.6 ± 1.9	2.7 ± 0.8	3.1 ± 0.9	3.0 ± 0.3	2.5 ± 0.8	2.3 ± 1.1
	/uL	12.0 ± 4.0	11.6 ± 3.3	15.5 ± 6.4	14.2 ± 1.4	13.9 ± 4.4	13.1 ± 7.1
Goblet cells	%	0.6 ± 0.2	0.8 ± 0.4	1.1 ± 0.6	1.2 ± 0.3	1.3 ± 0.2	1.1 ± 0.4
	/uL	2.7 ± 0.5	3.4 ± 1.4	4.3 ± 2.2	5.2 ± 1.3	7.0 ± 0.8	5.6 ± 2.4
Columnar epithelial cells	%	0.6 ± 0.2	0.3 ± 0.3	1.5 ± 0.6	0.9 ± 0.5	0.3 ± 0.2	0.2 ± 0.1
	/uL	3.4 ± 1.6	1.4 ± 1.4	7.2 ± 2.5	3.4 ± 1.6	1.7 ± 1.5	0.8 ± 0.6

Superscript letters (a–d) denote significant difference between time points, with, ^a^*p* = 0.0040; ^b^*p* = 0.0374; ^c^*p* = 0.0217; ^d^*p* = 0.0404; ^e^*p* = 0.0217.

When treated and control group means were considered individually and compared across three time points (*t* = 0, 6, and 24 h), there was a borderline significant difference between means (*p* = 0.0526) in the neutrophil percentage of control horses. Tukey's indicated a significant difference between *t* = 0 to *t* = 6 h, from 10.3 ± 1.6 to 16.9 ± 2.4 (*p* = 0.0188). Control group horses had significantly different mean percent macrophages with regard to time (*p* = 0.0024). Tukey's indicated that *t* = 6 h (40.0 ± 2.8) was significantly different than either *t* = 0 h (54.9 ± 3.2, *p* = 0.0012) and *t* = 24 h (53.1 ± 2.6, *p* = 0.0026). Both treatment and control groups demonstrated significantly different mean lymphocyte percentages between times (*p* = 0.0391, *p* = 0.0315, respectively). For both groups, means were highest at *t* = 6 h. Tukey's indicated that *t* = 6 h was significantly different than *t* = 24 h for the control group (*p* = 0.0108) and the treatment group (*p* = 0.0134).

**Figure 4:**
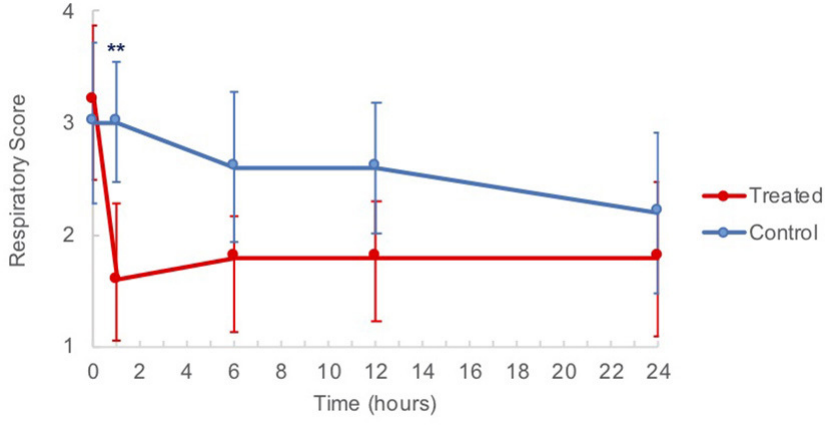
Clinical respiratory score means of horses from baseline (*t* = 0 h) to 24 h post nebulization with either control substance or G-CAPE (treated). **p = 0.0013.

Blood gas analysis did not differ significantly between groups (Supplementary Table 1). There was a significant difference (*p* = 0.0223) in the mean IDEASS respiratory scores of G-CAPE treated horses with regard to time. Tukey's indicated that baseline and 1 h post treatment means were significantly different (3.2 ± 0.7 to 1.6 ± 0.7, Figure 4). There was no change in scores of the control group from *t* = 0 h to *t* = 1 h. Across all post-treatment times assessed (*t* = 1–24 h), mean clinical scores remained lower for the treated group when compared to the control group.

A power calculation was done to determine the sample size needed to determine significant differences between times within groups. We assumed alpha = 0.05, power = 0.80, and estimated the effect size based on the definition (formula) of partial eta square. G^*^Power version 3.1 was used to then determine the sample size. For BAL neutrophil %, the necessary sample size = 11. For BAL total neutrophil count, the necessary sample size = 19.

## Discussion

The results of our study demonstrate that a single administration of nebulized G-CAPE resulted in improved clinical respiratory scores 1 h post treatment and reduced the percent neutrophils present in BAL fluid at 6 h after administration in healthy animals with induced airway inflammation.

The various clinical phenotypes of equine asthma have complicated understanding of the immunopathogenesis of the disease. NF-κB is a transcription factor that serves as an important regulator of inflammation. NF-κB induction of acute phase cytokines such as TNF- α, IL-1 and IL-6 direct recruitment and activation of inflammatory cells as well as other downstream events in the inflammatory cascade (30). As such, NF-κB has been associated with inflammatory gene expression in asthmatic horses, increasing at the onset of inflammation and persisting for up to 21 days after an allergen exposure (16, 31), and bronchial NF-κB has been strongly correlated to elevated bronchial neutrophil percentage (17, 32, 33). In the murine asthma model used by Jung et al. CAPE effectively suppressed NF-κB activity in the lung tissues (16). Taken together, these data suggest that inhibition of NF-κB by the specific pharmacological inhibitor G-CAPE has therapeutic potential. We hypothesized G-CAPE would inhibit NF-κB, resulting in a measurable mitigation of airway inflammation, including suppression of bronchial neutrophils. Due to the limited scope of this study, NF-κB and cytokine concentrations were not measured, but quantification would be recommended in future studies. Further investigation into the effects of repeated administration may yield more sustained effects.

The results of this study showed that administration of nebulized G-CAPE reduced mean BAL neutrophil percentages in horses 6 h after a single treatment. While it also did not reach statistical significance, the total neutrophil count also followed this trend; neutrophil count increased from *t* = 0 to *t* = 6 h in the control group and decreased in the treatment group. These data suggest NF-κB suppression thus resulted in suppression of bronchial neutrophil recruitment in the treated group.

The IDEASS respiratory scoring system utilizes a scale of 0–8 to classify asthma severity, with each one point change representing a minimally clinically detectable difference in clinical signs (2). The baseline score for all animals was similar (mean 3.2 ± 1.5 and 3.0 ± 1.6 for treatment and control, respectively), with an overall mean score of 3.1, correlating with moderate asthma. There was a significant improvement in score of the treated animals at 1 h post nebulization from 3.2 ± 0.7 to 1.6 ± 0.7 (*p* = 0.0013), while the scores of control horses remained unchanged over the same period. Although not statistically significant, the treated horses scored consistently lower at all post treatment timepoints. This demonstrated a measurable reduction in clinical signs and alteration in respiratory function after a single treatment. There were no significant changes measured on the arterial blood gas samples in this study; the pulmonary inflammation induced in otherwise healthy animals by our model was possibly not severe enough to overcome the reserve capacity of lung function and impair gas exchange.

The lymphocyte percentage in both the treated and control groups was increased in the *t* = 6 h BAL fluid sample; these samples represent 6 h post drug administration, as well as the second BAL procedure of the day. This is interesting, as acute lymphocytosis is not expected in the face of irritants or physical trauma. It is possible that the increased lymphocyte numbers are a reaction to triethanolamine, as some animal model safety studies have reported this phenomena to occur (while ultimately non-significant) (34). Another explanation may be that the particulate exposure protocol has created a model of a different form of chronic airway inflammation than that of typical moderate-severe equine asthma. In naturally affected horses exacerbated by environmental dust, airway neutrophilia and obstruction reliably occur. Although healthy horses are reported to respond variably to environmental challenged, we achieved a BALF neutrophil percentage >5%, which is the diagnostic criteria accepted in the ACVIM consensus statement, and therefore we achieved a reasonable degree of inflammation to treat (1, 5). The next step would be to explore this therapy in horses with naturally occurring asthma.

The increased neutrophil percentage in the control group at *t* = 6 h may be reasonably attributed to an inflammatory response to the iatrogenic trauma of the BAL procedure itself as has been described in previous studies (35–37). Considering this evidence and the increased neutrophilia seen in our controls, the simultaneous reduction in neutrophil percentage the G-CAPE treated animals may signify that the drug is both attenuating the pre-existing airway inflammation and blunting the response to the BAL procedure seen in the non-treated animals.

## Limitations

The degree of baseline inflammation induced in normal horses with the dusty hay described in some similar exposure models varied widely, with BAL neutrophil percentages ranging from 7 to 27% reported (38–42). This may reflect the variety of individual responses to airway challenge; horses affected with mild asthma present with several different phenotypes, resulting in differences in inflammatory cell populations found in BAL fluid (1). A larger sample size may have allowed us to overcome some of this variation or select for horses that had a greater neutrophilia in response to challenge. However, the mean baseline neutrophil percentage of 12.3% achieved is above the recognized minimum of >5% considered to represent neutrophilic airway inflammation (1).

## Conclusions

This study demonstrated that a single nebulized dose of the novel compound G-CAPE significantly decreased clinical respiratory scores 1 h post administration. There was also an apparent discordance in pulmonary neutrophil recruitment between treatment and control groups at 6 hours post treatment. These findings suggest that G-CAPE may reduce the pulmonary inflammatory response to inhaled particulate matter in horses.

Based on the results of this initial study, future studies should evaluate the pharmacokinetics, pharmacodynamics and anti-inflammatory efficacy of repeated G-CAPE nebulization. We believe that this compound shows promise as an anti-inflammatory and warrants further investigation. Studies in asthma-affected horses may yield a better understanding of this potential therapy.

## Data availability statement

The original contributions presented in the study are included in the article/Supplementary material, further inquiries can be directed to the corresponding author/s.

## Ethics statement

The animal study was reviewed and approved by Oklahoma State University Institutional Animal Care and Use Committee (Animal Care and Use Protocol IACUC-20-08).

## Author contributions

TH, JR, LG, JP, and JJR contributed to conception and design of the study. AS contributed to experimental material preparation. CM completed statistical analysis. EC contributed to data collection. All authors contributed to manuscript revision, read, and approved the submitted version.

## Funding

This study was supported in part by the Boehringer Ingelheim Animal Health 2019 Advancement in Equine Research Award.

## Conflict of interest

The authors declare that the research was conducted in the absence of any commercial or financial relationships that could be construed as a potential conflict of interest.

## Publisher's note

All claims expressed in this article are solely those of the authors and do not necessarily represent those of their affiliated organizations, or those of the publisher, the editors and the reviewers. Any product that may be evaluated in this article, or claim that may be made by its manufacturer, is not guaranteed or endorsed by the publisher.
