# Supplementary material for: Nebulized glycosylated caffeic acid phenylether ester attenuation of environmental particulate-induced airway inflammation in horses
Source: Front Vet Sci. 2022 Nov 3;9:958567. doi: 10.3389/fvets.2022.958567 (PMC9669659; doi:10.3389/fvets.2022.958567)
Supplement: Supplementary file 1 [file Data_Sheet_1.docx]

**Table S1.** Arterial pO_2_ (mm Hg) of horses from baseline (T = 0 hours) to 24 hours post nebulization with either control substance (C) or G-CAPE (Tx).

| **Horse** |  | **T=0** | **T=1** | **T=6** | **T=12** | **T=24** |
| --- | --- | --- | --- | --- | --- | --- |
| 1 | Tx | 108 | 117 | 92 | 90 | 96 |
|  | C | 85 | 91 | 86 | 108 | 96 |
| 2 | Tx | 73 | 88 | 74 | 74 | 98 |
|  | C | 90 | 86 | 90 | 78 | 91 |
| 3 | Tx | 83 | 93 | 83 | 108 | 88 |
|  | C | 67 | 94 | 86 | 98 | 101 |
| 4 | Tx | 100 | 95 | 93 | 92 | 91 |
|  | C | 103 | 113 | 89 | 112 | 96 |
| 5 | Tx | 110 | 107 | 102 | 102 | 109 |
|  | C | 118 | 110 | 88 | 102 | 108 |

**Table S2.** Respiratory scores of horses from baseline (T = 0 hours) to 24 hours post nebulization with either control substance (C) or G-CAPE (Tx). (Scores based on *Calzetta* et al*. EVJ 2018; 50(5):594-601.).*

| **Horse** |  | **T=0** | **T=1** | **T=6** | **T=12** | **T=24** |
| --- | --- | --- | --- | --- | --- | --- |
| 1 | Tx | 3 | 2 | 2 | 3 | 2 |
|  | C | 4 | 3 | 2 | 2 | 1 |
| 2 | Tx | 4 | 1 | 1 | 1 | 1 |
|  | C | 1 | 2 | 1 | 2 | 1 |
| 3 | Tx | 3 | 1 | 2 | 1 | 0 |
|  | C | 2 | 2 | 2 | 2 | 2 |
| 4 | Tx | 1 | 0 | 1 | 1 | 2 |
|  | C | 3 | 3 | 3 | 2 | 2 |
| 5 | Tx | 5 | 4 | 3 | 3 | 4 |
|  | C | 5 | 5 | 5 | 5 | 5 |
